# FlyBase: a guided tour of highlighted features

**DOI:** 10.1093/genetics/iyac035

**Published:** 2022-03-10

**Authors:** L Sian Gramates, Julie Agapite, Helen Attrill, Brian R Calvi, Madeline A Crosby, Gilberto dos Santos, Joshua L Goodman, Damien Goutte-Gattat, Victoria K Jenkins, Thomas Kaufman, Aoife Larkin, Beverley B Matthews, Gillian Millburn, Victor B Strelets, the FlyBase Consortium, Norbert Perrimon, Susan Russo Gelbart, Julie Agapite, Kris Broll, Lynn Crosby, Gil dos Santos, Kathleen Falls, L Sian Gramates, Victoria Jenkins, Ian Longden, Beverley Matthews, Jolene Seme, Christopher J Tabone, Pinglei Zhou, Mark Zytkovicz, Nick Brown, Giulia Antonazzo, Helen Attrill, Phani Garapati, Damien Goutte-Gattat, Aoife Larkin, Steven Marygold, Alex McLachlan, Gillian Millburn, Arzu Öztürk-Çolak, Clare Pilgrim, Vitor Trovisco, Brian Calvi, Thomas Kaufman, Josh Goodman, Pravija Krishna, Victor Strelets, Jim Thurmond, Richard Cripps, TyAnna Lovato

**Affiliations:** 1 Department of Molecular and Cellular Biology, Harvard University, Cambridge, MA 02138, USA; 2 Department of Physiology, Development and Neuroscience, University of Cambridge, Cambridge CB2 1TN, UK; 3 Department of Biology, Indiana University, Bloomington, IN 47405, USA

**Keywords:** FlyBase, model organism database, *Drosophila*

## Abstract

FlyBase provides a centralized resource for the genetic and genomic data of *Drosophila melanogaster*. As FlyBase enters our fourth decade of service to the research community, we reflect on our unique aspects and look forward to our continued collaboration with the larger research and model organism communities. In this study, we emphasize the dedicated reports and tools we have constructed to meet the specialized needs of fly researchers but also to facilitate use by other research communities. We also highlight ways that we support the fly community, including an external resources page, help resources, and multiple avenues by which researchers can interact with FlyBase.

## Introduction

FlyBase has a long and venerable history stretching back 30 years. During that time, we have grown into a dedicated team of curators and developers with an extensive level of experience and institutional knowledge. Over the past three decades, FlyBase has served as a crucial online resource to the Drosophila research community. We provide access to a wide variety of curated information from publications and large-scale projects, including genetic and genomic data, alleles and transgenic constructs, phenotypes, genetic and physical interactions, large datasets, and reagents for *Drosophila melanogaster*. FlyBase is continually developing and refining ways to concisely present, and allow researchers to easily access, a body of information that is ever-increasing, not only in quantity but also in scope and variety.

Like all major model organism databases (MODs), FlyBase offers a number of tools and reports that allow researchers to search and access data, and we have recently documented major updates to our knowledge base (Larkin *et al.* 2021). We have also developed a collection of tools and report types that are unique to FlyBase. In this study, we will highlight those aspects, many of which have required project-wide collaboration to bring to fruition, and some that have been the inspiration of one or two project members who have seen a need then undertaken a personal project to address that need. These features fall into two major categories: tools that help FlyBase users find the particular data they are looking for; and tools and reports in which FlyBase has organized data to make it more accessible.

In addition, we are taking the opportunity in this study to draw attention to some of FlyBase’s crucial but less flashy features. We support and interact with the Drosophila research community in many ways. We maintain a detailed external resources wiki, which provides access to a wealth of Drosophila information hosted outside of FlyBase, including links to cutting-edge technologies too new to be fully integrated into FlyBase. We provide the Fast-Track Your Paper tool, which allows authors to do partial self-curation of their publications, to get their data into FlyBase more quickly. FlyBase also offers a variety of user support resources, including extensive help documentation, a procedure for quickly answering user questions, a community advisory group, an archive of FlyBase publications and presentations, and Twitter-based tutorials.

## FlyBase tools to help find data

FlyBase users, like the users of all MODs, need to be able to find the particular data in which they are interested either by searching for data associated with a list of entities of interest (such as genes or publications) or querying curated data to generate lists of entities. In this section, we present some tools developed at FlyBase to assist our users in their data quests.

### QuickSearch

**Fig. 1.**
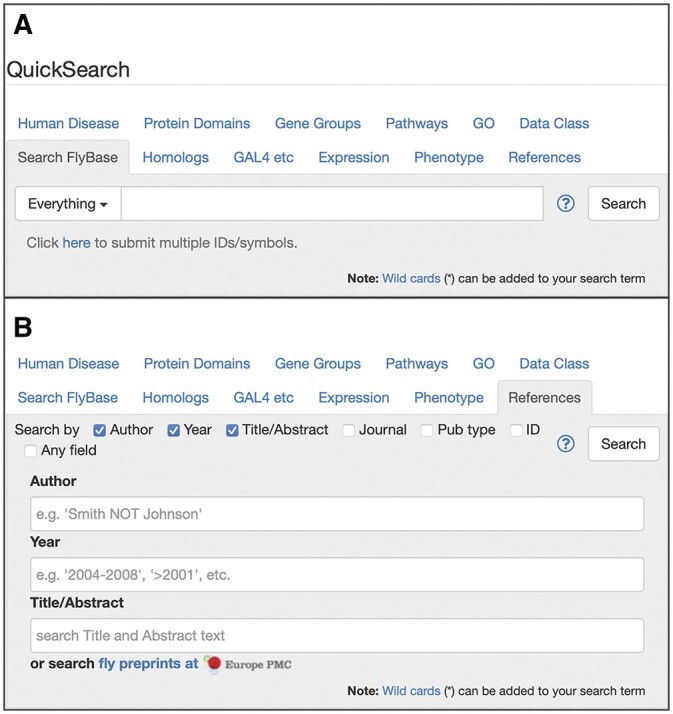
a) The FlyBase QuickSearch tool, a collection of a dozen search tools. The “Search FlyBase” tab supports uploading a list of multiple IDs or symbols into the search. b) The “References” QuickSearch tab. In addition to searching the FlyBase bibliography, this tab includes a link to search fly preprints at Europe PMC.

QuickSearch, located prominently in the center of the FlyBase homepage, is the search hub for querying data. Originally, it was a quite simple search tool, consisting of a single text box and a pull-down menu from which one could choose a data class to query (Wilson *et al.* 2008); this was later enhanced by the addition of tabs dedicated to the search of specific data classes (McQuilton *et al.* 2012). The current version of QuickSearch provides a broad spectrum of search tools for interrogating Drosophila data in FlyBase, including a basic full-text search, publications, expression, and much more (Fig. 1). Each tool is organized into a tab that is labeled by the data domain on which the tool specializes. Most tabs make use of ontologies and controlled vocabularies (hierarchies of terms that are linked to each other in defined ways) and have an autocomplete feature. Some tabs have additional functionality, such as links to related tools, resources, or lists. The dozen tabs that currently comprise QuickSearch are described in Table 1 [for more detail see Jenkins *et al.* (2022) and Marygold *et al.* (2016)].

**Table 1.** The QuickSearch tool

Quick Search tab	Query supported by tab
Search FlyBase	Searches nearly all data; can be used to search either free text, or be limited to search only FlyBase identifiers, symbols, and names. Also supports searching with multiple IDs or symbols, or an uploaded file.
Expression	Searches by temporal and spatial expression pattern of genes using controlled vocabulary terms for stage, anatomy, and/or cellular component. Also includes links to tools that allow one to search or browse RNA-Seq high-throughput expression data.
GAL4 etc	Searches by temporal and spatial expression pattern for transgenic reporters such as lacZ or GFP, or binary drivers such as GAL4 or lexA; also supports searching for transgenic drivers and reporters that reflect the expression pattern of a specific gene. Also links to the Frequently Used GAL4 Drivers resource, which displays compiled information for over 300 popular drivers.
Gene Groups	Searches for Gene Group reports; searches can use Gene Group symbols, names, synonyms, or identifiers, or component genes in a Gene Group. Includes a link to a browsable list of all Gene Group reports.
Pathways	Searches for Pathway reports; searches can use Pathway symbols, names, synonyms, or identifiers, or component genes in a Pathway Includes a link to a browsable list of all Pathway reports.
Protein Domains	Searches for genes whose products contain a specific protein domain, repeat, or site using InterPro identifiers or signatures (Blum *et al.* 2021).
GO	Searches for Gene Ontology terms (Gene Ontology Consortium 2021); searches can be of all GO terms, or can be limited to molecular function, biological process, or cellular component terms.
Phenotype	Searches for alleles that have a particular phenotype; searches can be by phenotypic class and/or by phenotype affecting a specific tissue or cell type.
Homologs	Supports searches of orthologs to human, *Drosophila melanogaster*, and 8 other model organism genes, and paralogs to Drosophila genes using DIOPT (Hu *et al.* 2011) which integrates multiple orthology prediction algorithms.
Human Disease	Searches for HDM reports, DO terms (Schriml *et al.* 2019), disease model-associated genes, and alleles annotated with DO terms; search terms can include any of those, as well as OMIM terms or disease synonyms (Amberger *et al.* 2019). Includes a link to a browsable index of all HDM reports.
References	Searches the FlyBase bibliography; search can be by any combination of author, year, title/abstract, journal, publication type, or identifier (FBrf, PMID, PMCID, DOI). Includes a link to fly preprints at Europe PMC.
Data Class	Supports searches restricted to a single data type, chosen from a pull-down menu of over 30 data types, from Aberrations to Transgenic Constructs; this tab is similar to the original QuickSearch tool.

Each QuickSearch tab supports a specialized query function. Some tabs include links to additional resources or tools.

### Interactive HitLists

Most of the FlyBase search tools return their results in an interactive format called a HitList. The HitList is an important central hub for searches on FlyBase that allows users to view, filter, export, and analyze results (Thurmond *et al.* 2019). Understanding what a HitList is and what it can do is critical to making the most efficient use of a user’s time on FlyBase.

There are multiple ways to start working with a HitList. The most common way is to perform a search with a tool such as QuickSearch. Once the search has completed, your results are displayed in a HitList. A HitList can also be generated by uploading via the ID Validator tool and many other tools and reports offer an “export to HitList” option (Larkin *et al.* 2021).

**Fig. 2.**
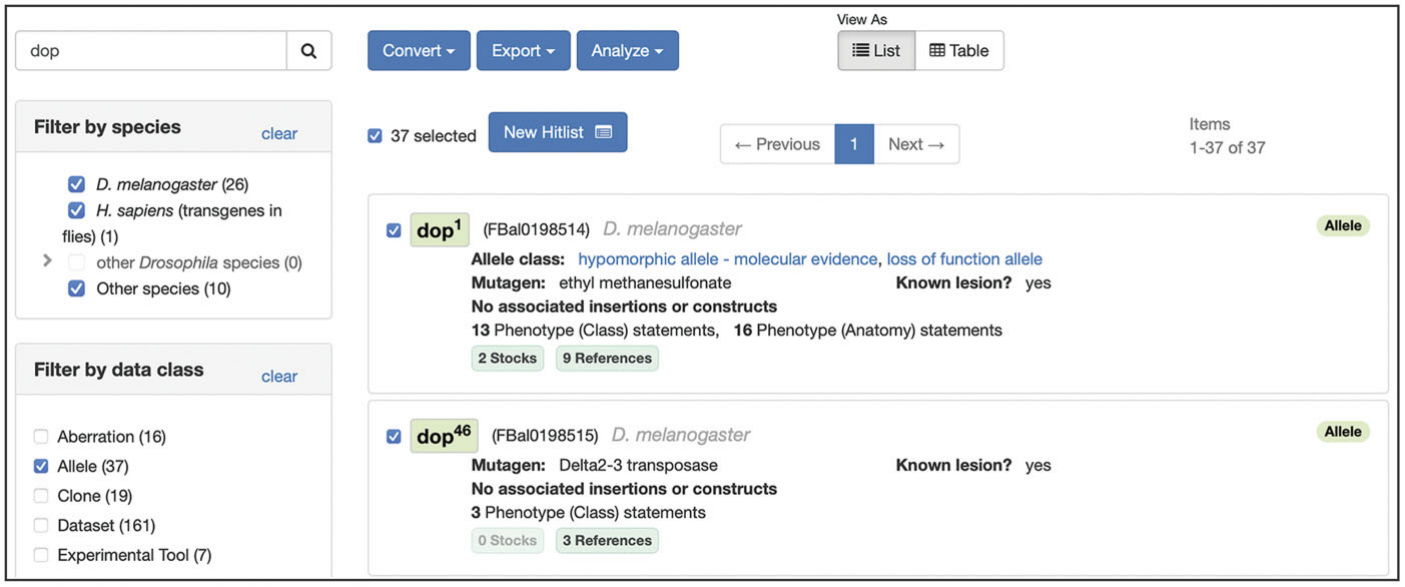
The FlyBase HitList. A HitList resulting from a search for the text string “dop” has been filtered by the data class “Allele.” The filtered HitList, which includes alleles from multiple species, can now be viewed as a table, or further manipulated using the “Convert,” “Export,” or “Analyze” button menus.

The HitList enables users to refine the list of results by selecting species and data class—particularly important when dealing with a mixture of FlyBase objects/CV terms e.g. alleles, genes, fly anatomy ontology terms (Fig. 2). Selecting a single data class allows more complex operations to be performed, such as converting between linked data classes (e.g. Genes to Stocks), exporting to other FlyBase tools or as a downloadable list, or analyzing co-occurring annotation data such as Gene Ontology (GO) terms, expression stage or genetic interaction graphs. In addition, for a single data class, the HitList can be viewed as a sortable table and entities can be manually selected allowing further refinement of the set.

### Responsive tables

FlyBase, like most database resources, includes tabulated data in report pages. As the types of information that can be associated with alleles, insertions, constructs, and pathways have proliferated, corresponding columns have been added. As a result, data tables are often wider than browser screens can accommodate, especially when accessed on a mobile device. However, narrowing a static table by removing columns also removes information important to many users.

**Fig. 3.**
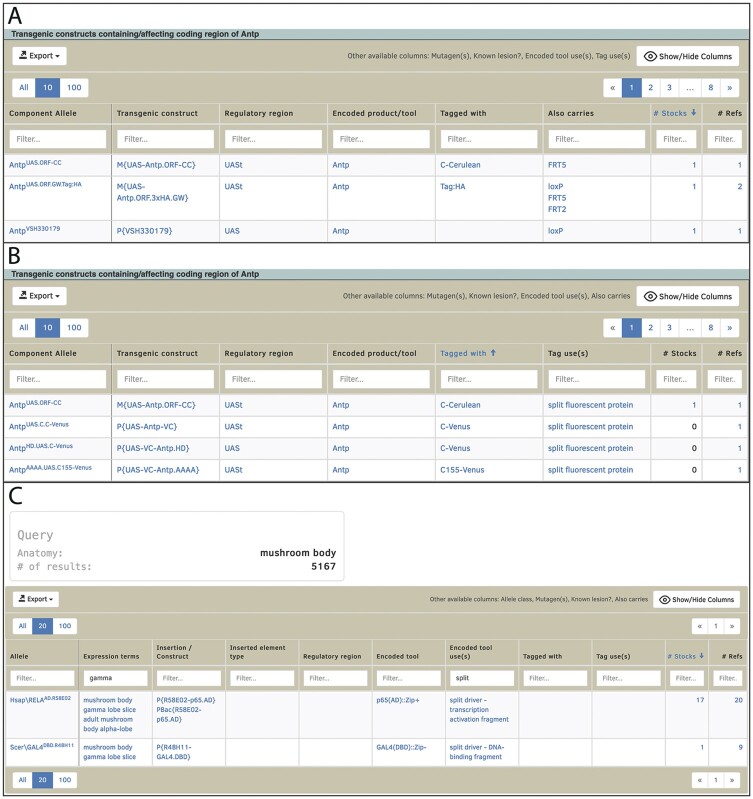
a) The responsive table includes options to show, hide, or sort columns; hidden columns are displayed next to the Show/Hide Columns button. b) The responsive table shown in panel (a) has been customized, sorting the table by the “Tagged with” column (bringing tagged transgenic constructs to the top of the list), showing the previously hidden “Tag uses” column (to provide information about the nature of the tag), and hiding the “Also carries” column. c) The “GAL etc” QuickSearch HitList uses responsive tables to filter HitLists that can initially be very large. In this case, a search for transgenic reporters expressed in the mushroom body has over 5,000 hits. The list has been narrowed by filtering the “Expression terms” and “Encoded tool use(s)” columns to show only split-GAL4 drivers expressed in the gamma lobe of the mushroom body.

Responsive tables solve this issue by allowing users to narrow down lists of related data to the particular subset they are interested in, since the table can be customized by the users to show, hide or reorder individual columns (Fig. 3, a and b) and filters can be applied to multiple columns to narrow down the results to rows matching the filters (Fig. 3c). The filtered results can then be downloaded or exported to a FlyBase HitList for further interrogation (Larkin *et al.* 2021). Responsive tables are currently implemented for lists of related alleles, insertions and transgenic constructs (on Gene reports, the Frequently Used GAL4 Drivers resource and in results of the “GAL4 etc” QuickSearch tab) and for the members table on Pathway reports.

## FlyBase reports and tools that organize data

Searches are not the only way that FlyBase users can access data of interest. Some groups of data are presented in dedicated reports that organize that information to make it easily accessible. Dedicated reports described below include those for gene groups and pathways, human disease models, reagent collections, and metadata for high-throughput datasets. We have also developed a new data class to make it possible to identify genetic tools with defined molecular properties. Finally, we display many high-throughput datasets in JBrowse, including a stunning visualization of RNA-seq data.

### Gene groups and pathways

Gene sets are useful—a list of genes related in some way may provide the starting point for a genetic/molecular screen or in silico analysis, or allow comparison with equivalent gene sets in other species. The rationale behind the manual curation of Gene Groups and Pathways is to provide gold-standard sets of genes, presented in dedicated report pages (Attrill *et al.* 2016), collated and reviewed by FlyBase in a systematic manner to ensure that the set is as accurate, complete and up-to-date as possible. Alongside the collation of these sets, we review the associated GO (Ashburner *et al.* 2000; Gene Ontology Consortium 2021) annotation to ensure that genes are annotated comprehensively with terms describing their function with respect to the expected set of biological characteristics of the set e.g. kinases are labeled with the GO term “kinase activity” (GO:0016301) or its child terms. This improves the accuracy of gene sets generated by a GO query or by using the Vocabularies tool.

Gene Groups in FlyBase encompass several types of gene/gene product sets including macromolecular complexes (e.g. “ATAC COMPLEX”), sets related by common molecular function (e.g. “DEUBIQUITINASES”), and sets related by a biological role (e.g. “CUTICLE PROTEIN FAMILIES”). Pathway reports have been constructed for 16 key signaling pathways, including 6 receptor tyrosine kinase pathways and other major developmental pathways such as Hedgehog signaling. Pathway and Gene Group reports are organized in a hierarchical fashion, usually under a grouping parent report. For Pathways, there is very little nesting—a top-level parent report with the sub-groups: “core” (those genes that are required for the functional pathway), “positive regulators” and “negative regulators” and, in some cases, a “ligand production” report. In contrast, some gene groups have multiple tiers of organization. For example, the largest gene group, “ENZYMES,” is broken down into the major functional sets: “OXIDOREDUCTASES,” “TRANSFERASES,” “HYDROLASES,” “LYASES,” “ISOMERASES,” “LIGASES,” and “TRANSLOCASES.” These are further subdivided into smaller functional sets, such that the terminal groups give the most informative level of granularity e.g. “NUCLEOSIDE DIPHOSPHATE KINASES.” Thus, the user can determine the scope of the set they select.

Gene Groups are curated from a variety of sources: reviews, research publications, external databases, and in the case of enzymes, bioinformatic data-mining. The curation of Pathway reports is somewhat different, aimed at reflecting the research landscape. For these reports we redundantly curate experimental evidence from different research papers, using defined criteria for pathway inclusion. The number of papers that provides direct experimental evidence for a gene’s role in a pathway are displayed in the table with the aim that this information should help users differentiate between a novel regulator and a well-documented central pathway component, for instance. As pathway research is very active, these pages are updated to keep them current and new pathway members can be flagged by users in the Fast-Track Your Paper tool. With the additional aim of facilitating pathway research, we also generate a visual representation of experimental pathway knowledge, combining our pair-wise physical interaction data with an evidence-weighted display of members to generate an interactive network. The description is accompanied by a pathway thumbnail image—showing a more simplified, text-book style representation of the pathway, which can be used as an orientation aid when looking at the table or network or can be downloaded and modified for use in publications or talks, for example.

### Human disease models

A growing number of human diseases have been modeled in *Drosophila*: the current FlyBase release includes over 1,100 specific disease models described in over 4,300 primary publications. The human disease model (HDM) report is designed to bring together information concerning a modeled disease (Millburn *et al.* 2016). Rather than providing a comprehensive summary, the HDM report serves as an information hub combining FlyBase-specific curation with extensive links to medical database content and a comprehensive list of Drosophila-related references relevant to the disease, including primary publications, commentaries, and reviews. OMIM (Online Mendelian Inheritance in Man; Amberger *et al.* 2019) serves as a primary source of information on disease phenotypes and genetics for disorders with a genetic basis. However, a significant subset of Drosophila disease models do not fit neatly into OMIM categorizations and/or are not tractable to Disease Ontology (DO) (see below) annotation of genes or alleles. Many human diseases with a nongenetic basis are studied extensively in flies, such as traumatic brain injury, chemically induced disease phenotypes, diet-induced phenotypes, introduction of pathogens, and stress-induced behaviors. In addition, some models, including many covering the cutting edge of Drosophila translational research, fall into the category of postulated gene-disease associations and are not yet included in OMIM or other disease databases. The HDM report format allows presentation of information related to such nongenetic and preliminary models of disease.

At the top of the HDM report are brief summaries of the fly model, human disease phenotypes, known genetics of the disease, synonyms of the disease, and known functions of the implicated gene(s). Links to related reports in external resources are provided, including MedGen and MedlinePlus (NCBI Resource Coordinators 2018), KEGG pathways (Kanehisa *et al.* 2017), COSMIC (Tate *et al.* 2019), and SFARI Gene (Banerjee-Basu and Packer 2010). The related diseases section includes links to other HDMs that share a common molecular mechanism or causative gene, are related postulated diseases, or are general disease model reports covering reviews and methods. If the disease is part of an OMIM phenotypic series (grouping subtypes of one disease), links to OMIM and to other Drosophila models within the series are provided.

In subsequent sections, links to HGNC (Tweedie *et al.* 2021) and FlyBase Gene reports provide information on human and Drosophila genes used in the context of the disease model. If the human gene has been transgenically expressed in flies, a link to the FlyBase Gene report is included; nonhuman mammalian, synthetic, bacterial, or viral transgenes used in disease models are similarly documented and linked.

Details of genetic models of human diseases are captured at the allele level, using controlled terms from the DO (Schriml *et al.* 2019). For each gene linked to an HDM report, all alleles associated with a DO term are shown in a tabular presentation; a similar table is presented in the Gene report for the relevant gene. An important component of the DO annotation is capturing genetic interactions that modify the original disease phenotype, i.e. alleles of other genes that exacerbate or alleviate the disease model allele. The presentation of DO data on the HDM report provides a compact view of all the associated DO annotations. Tables of alleles used or potentially useful for modeling a human disease and links to stocks available at the public stock centers are provided.

Human variants are known to cause a specific disease are frequently used to model the disease in flies, using either a transgenic human gene or an analogous mutation in the orthologous Drosophila gene. A growing body of work makes use of *Drosophila* to characterize newly discovered human variants that are postulated to be associated with disease. In HDM reports, alleles described as representing disease-implicated variants are highlighted in a table that groups alleles analogous to the same human variant. Designations using standardized HGVS nomenclature (den Dunnen *et al.* 2016) and links to ClinVar (Landrum *et al.* 2018) are included to facilitate comparison of comparable alleles and to provide descriptions compatible with those used by the medical community. To date, approximately 370 Drosophila disease models in FlyBase include characterization of disease-implicated variants.

FlyBase provides multiple routes for finding a HDM report of interest, starting with the QuickSearch “Human Disease” tab (Table 1). Within the HDM report itself, there are links to related diseases; this is particularly useful for finding members of a phenotypic series. Gene reports include links to the HDM report within the “Human Disease Associations” section. Allele reports include a link in the “Disease-implicated variant(s)” subsection of the “Human Disease Associations” section, where appropriate. Finally, FlyBase DO Term reports include a link to HDMs curated to that term.

### Experimental tools

The long history of fly research, plus the sophisticated range of applicable genetic engineering techniques, mean that a large number of increasingly complex transgenic fly lines have been generated and described in the literature. While this rich genetic toolkit helps to make *D.* *melanogaster* an ideal model organism to answer a wide range of biological questions, it also creates a challenge—how to find the most appropriate fly line for a particular experiment from the large set that is available. To help address this, FlyBase has introduced an “experimental tool” data class.

An experimental tool in FlyBase is broadly defined as a commonly used sequence with useful properties that are exploited to study the biological function of another gene product or a biological process. These include tools that enable a gene product to be detected (e.g. the FLAG tag, EGFP, mCherry), target a gene product somewhere specific within a cell (e.g. nuclear localization signal), drive expression in a binary system (e.g. UAS, GAL4), enable clonal/conditional expression (e.g. FLP, FRT), molecular sensors (e.g. pH sensors), genome engineering tools (e.g. RNA-guided nucleases, integrases) and tools that modify cellular activity (neuron activation/inhibition tools).

**Fig. 4.**
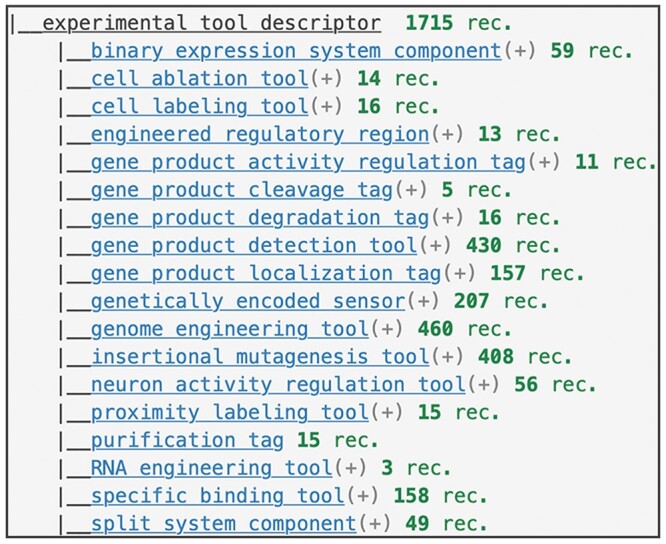
Main branches of the controlled vocabulary tree used to describe “Uses” of experimental tools. Term report for experimental tool descriptor (FBcv: 0005001) with children set to a hierarchy level of 1.

For each experimental tool, controlled vocabulary terms are used to describe its common use (e.g. epitope tag, green fluorescent protein, recombinase) (Fig. 4). Currently, over 39,000 alleles representing transgenic constructs and over 9,700 insertional alleles are labeled as encoding an experimental tool.

When a new allele or transgenic construct is described in the literature, any relevant experimental tools will be linked to the allele/transgene made in FlyBase, building up a picture of the components of which it is made (Table 2). This component information is included in the “responsive tables” of transgenic constructs and insertions (described above and shown in Fig. 3), so that users can easily identify fly stocks that have the particular characteristics they are interested in.

**Box 1. FlyBase in the classroom.**

FlyBase is a one-stop shop for teaching basic to complex biological concepts while integrating these concepts with bioinformatics instruction. A key element of FlyBase that makes it a unique resource for the classroom is its depth and breadth of available data compiled from multiple sources that is accessible to both beginners in the form of Gene reports as well as more advanced students with its array of query and analysis tools. Moreover, FlyBase lends itself well to curricula that begin in the laboratory and use FlyBase to analyze the resulting data, begin by using FlyBase data to make predictions that are tested in the laboratory, or are completely informatics-based.

Instructors at all levels looking to incorporate hands-on Drosophila modules into their laboratory classes may find the open access e-book, Experiments with Drosophila for Biology Courses, which FlyBase recently began hosting at http://flybase.org/commentaries/2021_04/LakhotiaRanganathEBook.html (last accessed 03 March 2022), particularly useful. This book, edited by S.C. Lakhotia and H.A. Ranganath (Lakhotia and Ranganath 2021), contains 85 laboratory exercises contributed by fly researchers that are designed to teach many fields of biology using the limited facilities available in many high schools and universities. For example, one classic genetics exercise includes performing dihybrid crosses to teach the ideas of Mendelian inheritance, linkage, and epistasis. FlyBase can be used by students to ascertain the parental genotypes based on phenotypic ratios of the progeny if the parental genotypes are unknown or predict phenotypic ratios of the progeny when the parental genotypes are known.

For bioinformatics-based modules, FlyBase can be used to generate or evaluate gene lists, make predictions based on the extracted information and test the predictions with further bioinformatic analysis and/or in the laboratory. For instance, the regulation of gene expression can be explored by generating a list of genes with similar expression patterns using FlyBase’s RNA-Seq Expression Profile Search tool with the expectation that the expression of such genes may be under the control of a similar set of transcription factors. Upstream sequences likely to contain regulatory elements for these genes can be downloaded using FlyBase’s Sequence Downloader tool and be exported to external tools for transcription factor binding motif prediction.

Finally, classroom exercises in which students use FlyBase to investigate orthology between Drosophila and human genes and pathways can serve to highlight the degree of evolutionary conservation between humans and flies. Each FlyBase Gene report contains a human orthologs section that is powered by DIOPT, the DRSC Integrative Ortholog Prediction Tool (Hu *et al.* 2011), which integrates multiple orthology prediction algorithms to provide an overview of potential orthologs. Such exercises further highlight the importance of the fruit fly as a tool for understanding basic human biology as well as for gaining insights into human disease.

This section is based on discussions with Alder M. Yu of the University of Wisconsin-La Crosse, Wilson Leung of the Genomics Education Partnership, Washington University-St. Louis, Martha Bhattacharya of the University of Arizona, and Brian Dempsey of Acton-Boxborough Regional High School.

**Table 2.** Linking experimental tools to a transgene/allele to describe its component parts.

Type of linking	Description	Example
Regulatory region	The tool corresponds to an engineered regulatory region which is used to drive expression of the gene product encoded by the transgenic construct/modified endogenous locus. (*If the regulatory region is a gene, rather than an experimental tool, that gene will be added in the regulatory region slot.)	UAS, 3xP3
Encoded tool	The entire gene product encoded by the transgenic tool construct/modified endogenous locus acts as a tool.	Cas9, EGFP
Tagged with	The tool is fused to (“tags”) another gene product whose biological function is being studied, and confers a novel property on that gene product.	Tag: HA, mCherry
Also carries	The tool does not form part of the gene product, but is “carried in” the transgenic construct/modified endogenous locus.	loxP, FRT

Tools can be linked to transgenic constructs, and to alleles generated by insertional or targeted mutagenesis (e.g. CRISPR) of the endogenous locus. Four different types of links (first column) can be made between the tool and each individual allele/transgene, building up a formalized description of its components.

### Large-scale datasets and reagent collections

The sheer number of high-throughput datasets poses challenges not only in processing data at large scale, but also in providing that data to users in such a way that data of specific interest can be easily found and used. As such, FlyBase has taken a selective approach to the incorporation of large-scale datasets. This is not only a response to the practical limitations of how much data we can process, but also a deliberate curation decision to keep our web pages streamlined.

Our strategy has been to identify the datasets that are of the broadest interest to our community, to focus on bringing in the high-level summary data (rather than raw data) that is most easily interpreted at a glance, to integrate that new data with other aspects of FlyBase as much as possible, and to provide dataset reports that describe the experimental methods with links to related publications and data repository accessions for those interested in digging deeper.

The datasets chosen are typically “reference” datasets that describe some aspect of biology in wild-type *Drosophila*, often across a broad range of developmental stages and/or anatomical tissues. Prominent examples include expression datasets from modENCODE (Graveley *et al.* 2011; Brown *et al.* 2014), FlyAtlas (Chintapalli *et al.* 2007; Leader *et al.* 2018) and a developmental proteome (Casas-Vila *et al.* 2017), and variation data from the DGRP reference strains panel (Mackay *et al.* 2012), protein–protein interactomes (Guruharsha *et al.* 2011; Rhee *et al.* 2014; Shokri *et al.* 2019), and DNA-binding site data from ChIP-chip/ChIP-seq datasets from modENCODE and the Furlong lab (Negre *et al.* 2011; Junion *et al.* 2012).

Before data incorporation, we curate the dataset metadata to create dataset reports. These reports cover various aspects of the dataset: biosamples, assays (raw data), various results (processed data), and a “project” report that groups all of those things together. These dataset reports provide detailed information on the methods used, links to relevant publications, data files for download, and data repository accessions for raw data. These reports also link all of that metadata to the actual data that we eventually incorporate, making it easy to dig into the methods and raw data behind any of the results that we display at FlyBase.

The way in which high-throughput data are incorporated depends on the data type, and can involve several approaches that involve the JBrowse genome viewer (Buels *et al.* 2016), custom gene page data visualizations, download files, and sometimes even custom data analysis tools. RNA-seq genomic coverage profiles are displayed in JBrowse using our novel “TopoView” visualization, a compact presentation of RNA-seq profiles that facilitates comparison between samples of a set (see below for details on the development and implementation of this novel display). For datasets consisting of discrete features (like transcription factor binding sites or exon junctions), we provide a standard JBrowse track that depicts the genomic extent of each feature; clicking on a feature in JBrowse opens the FlyBase report for that feature, which provides more detailed information on the feature, including experimental metadata from the associated dataset reports. For datasets that provide gene expression data across various developmental stages and tissues, like modENCODE RNA-seq (Graveley *et al.* 2011; Brown *et al.* 2014), FlyAtlas microarray (Chintapalli *et al.* 2007; Leader *et al.* 2018), and the developmental proteome (Casas-Vila *et al.* 2017), we have created gene expression histograms on our Gene reports (Box 1). We have also developed a number of custom tools to help users search and analyze the modENCODE RNA-seq dataset. The “RNA-Seq Profile” tool allows users to find genes that have a given expression profile: for example, present in adult ovary, but not in adult testis. The “RNA-Seq Similarity” tool allows users to find genes that share a similar expression profile to a given gene of interest. The “RNA-Seq by Region” tool provides RNA-seq signal across tissues or development for any specified region.

**Fig. 5.**
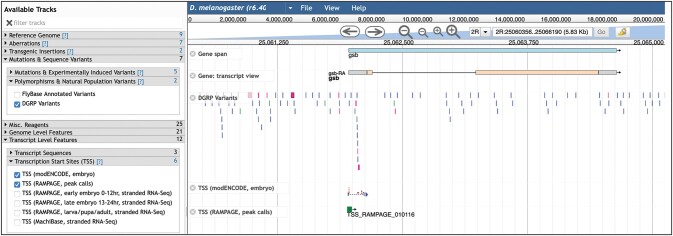
High-throughput data displayed in JBrowse. This view shows DGRP variant data, and two sets of transcription start sites.

We take a similar approach when incorporating large-scale reagent collections, an extremely high priority for FlyBase. We generate reagent collection reports (similar to dataset reports), which group together reagents of a collection and associate those reagents with pertinent metadata (biological samples, experimental methods). Where possible, we create JBrowse genome viewer tracks that allow users to assess these reagents in a genomic context (relative to other reagents or biological features of interest) (Fig. 5). Reagents are prominently displayed in the relevant sections of Gene reports. Examples of reagent collections include cDNA collections (Rubin *et al.* 2000), dsRNA transgenic fly collections (Dietzl *et al.* 2007; Ni *et al.* 2011), GAL4 driver collections(Pfeiffer *et al.* 2008; Kvon *et al.* 2014), and sgRNA transgenic fly collections (Jia *et al.* 2018; Meltzer *et al.* 2019).

### Enhanced display of RNA-seq data: TopoView

With the advent of the high-throughput modENCODE RNA-Seq data, FlyBase sought new ways to integrate this valuable global transcriptomic information. The modENCODE project had produced transcriptional profiles of multiple developmental stages, dissected body parts, chemical treatments, and several different tissue culture lines. These data were incorporated for search and display on a gene-by-gene basis amongst these different developmental times, tissues, and environmental conditions. FlyBase additionally developed a tool to allow users to view expression profiles in the context of FlyBase annotated gene models. This type of display of the RNA-Seq data would permit a quantitative comparison of levels of mRNA expression and alternative RNA splicing and promoter usage at different times, places, and conditions. The most convenient place to create a visual comparison of gene structure and expression is in JBrowse, where different tracks show gene models and RNA transcripts. We created tracks that showed the level of RNA expression aligned with the genomic positions in the browser. While mRNA signal graphs had been adopted previously, the creation of separate tracks for each data set was not readily scalable and the comparison of data from different tracks was difficult. This problem was compounded as the number of separate profiles (samples, specimens, etc.) grew to several hundred and even thousand, with the number of desired comparisons being quite large (multiple developmental stages, several different body parts, etc.). We thus developed a compacted view of multiple tracks for the RNA-Seq data/profiles, which can be used for comparison of gene expression in different samples facilitating a visual comparison of different expression signal profiles by the user. We named this new configuration TopoView.

**Fig. 6.**
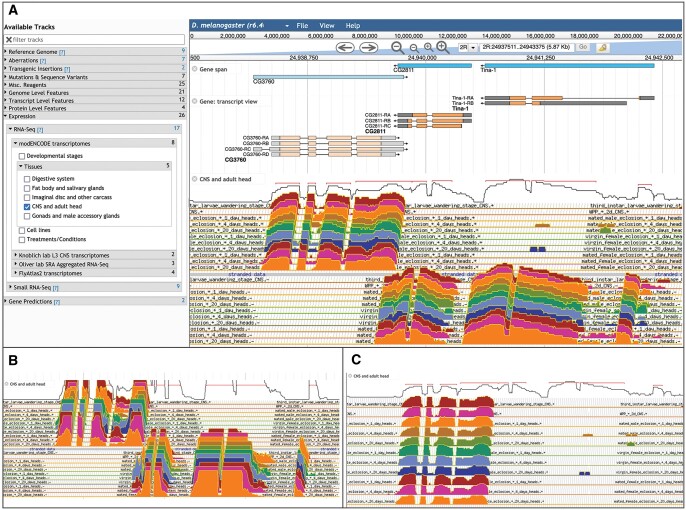
a) TopoView. This view shows stranded RNA-seq data, in the default view using a log2 signal scale and a tilted, tightly spaced display. Each colored track represents a specific developmental time point, tissue, and/or sex from which samples were collected. b) Customizable TopoView display options. This view shows the same data as in panel (a) displayed using a linear signal scale. c) This view displays the same data using a log2 signal scale with increased vertical spacing, making it easier to see the tracks at the rear.

TopoView allows the presentation of groups of profiles in a single view with configurable options to aid in RNA-Seq data visualization (Fig. 6a). This serves several purposes: First, it creates a very compact presentation of samples/specimens in the form of groups of overlapping profiles which can dramatically decrease screen space used for viewing (e.g. FlyBase shows 30 developmental stage samples as a single track). Second, it allows for easy and intuitive comparison of signals. And finally, it allows the user to toggle profiles between tilted (topographical style) or vertical presentation, log-scaled signal *vs.* linear, the ability to adjust vertical spacing (overlap) of profiles, and selection of subsets (samples/specimens) to view from profiles included in the track (Fig. 6, b and c). TopoView has contributed to a new appreciation of genome organization and expression, including that many genes produce different mRNA isoforms during development and in response to environmental conditions.

To make the powerful TopoView tool available to other genomic databases and MODs, FlyBase developed TopoView as an open source JBrowse plugin to display continuous signal data as a chromosome-wide mosaic. To generate the mosaic, the client-side plugin interacts with a server-side component, which dynamically generates pieces of the mosaic for the selected genome coordinates. At FlyBase, the server-side application uses Perl and the GD graphics library to render the image segments. However, any programming language and graphics library that conforms to a predefined set of parameters expected by TopoView can be used if desired. The client side TopoView plugin follows the standard JBrowse configuration style used by many other plugins. In addition, the TopoView plugin is not limited to RNA-Seq data and can be used for other types of genome-wide continuous features. For example, FlyBase uses it for displaying chromatin domain data. Given this versatility, TopoView has the potential for the display of different types of data in multiple organisms to facilitate biological discovery.

## Community and external resources

### External resources

FlyBase strives to give users access to everything *Drosophila*-related. Besides the content on our own site, FlyBase actively maintains External Resource wiki pages with links to hundreds of useful Drosophila online resources. The External Resources wiki can be accessed from the Tool/Reagent Resources (wiki) button in the left sidebar, on the home page or from the “Links” dropdown menu at the top of every page.

The External Resources wiki page is divided into two main sections. At the top is the “Popular Resource Categories” section with large buttons that take you to dedicated pages for a variety of topics. These include pages on popular techniques (CRISPR, scRNA-seq, and RNAi), pages with links to help find useful reagents (Stocks and Antibodies), and popular topic-specific pages (Neuroscience, Images, Maps). The “MODs” button leads to links to all the major MODs as well as a link to the Alliance of Genome Resources. The “Protocols” button takes you to a sorted list of protocol-relevant publications created by the Drosophila RNAi Screening Center (DRSC; Hu *et al.* 2021). Finally, the new “Papers with Technical Advances” page displays papers that have been identified by users during the Fast-Track Your Paper process (see below) as presenting a notable technical advance, new reagent or resource likely to be useful to other researchers. Dedicated Wiki Resource pages are sometimes used as a first step to give users access to popular new data types that are in the process of being incorporated into FlyBase. For example, the Resource page for the emerging technology of single-cell RNA sequencing (scRNA-seq) provides links to scRNA-Seq data portals, a variety of scRNA-seq data analysis tools, and the Fly Cell Atlas consortium (Li *et al.* 2022).

Further down the page, in the “All Resources” section, hundreds of links to *Drosophila*-relevant websites have been divided into two groups. The “Online” resource section contains links to information-based sites, including atlases and images, HDMs, and uses of *Drosophila* in public education. The “Material Resources” reagent section offers sources for a variety of DNA reagents and cell lines, as well as links to transgenic and mutagenesis services. FlyBase always welcomes suggestions for additional links to add to our catalog.

### FlyBase community advisory group

While working to improve the data in FlyBase, questions arise about which data are the most important for Drosophila researchers and how we can most usefully present those data. We set up the FlyBase Community Advisory Group (FCAG) in 2014 to consult our user community as needed (Thurmond *et al.* 2019). The FCAG currently comprises 880 representatives from 45 countries. We ask FCAG members to take 4–6 short surveys per year. Not only does this allow us to make decisions that will benefit the greatest number of users, but often FCAG participants discover a feature in FlyBase that they did not know about and can share with others.

### Fast-Track Your Paper

The Fast-Track Your Paper (FTYP) tool was first introduced to FlyBase in 2009 (Bunt *et al.* 2012) and has undergone various upgrades, most recently in 2020 (Larkin *et al.* 2021). The tool enables first-pass curation by authors, allowing association of relevant genes to their reference and indicating to FlyBase curators what classes of data are in the study, which accelerates incorporation of relevant data into FlyBase, thus benefiting both users and curators. Though the tool can be accessed at any time via the Tools menu bar, most authors will use FTYP after receiving an e-mail from FlyBase, informing them that their recent publication is now in FlyBase, and encouraging them to add their data. Currently, around 40% of authors who are contacted submit data from their publication via the FTYP tool. Four data types (disease, new allele, new transgene, physical interaction) are automatically text-mined by FlyBase (Fang *et al.* 2012; McQuilton and FlyBase Consortium 2012) when a paper enters our database, and those results are pre-populated in the tool if detected. Authors can check that these have been correctly detected, and additionally indicate other relevant data types (e.g. cell line used, gene rename, initial or novel characterization of a gene, expression data, phenotypic data, genome annotation data). Authors can also indicate if there is a technical advance and provide a short description of it; papers so flagged are added monthly to the “Papers with technical advances” FlyBase resource page.

## User support resources

An aspect of MODs that often goes unrecognized is the ways in which the curation and developer teams support users. FlyBase has developed substantial user support resources, including multiple varieties of help documentation, a robust help mail procedure, lists of our publications, and archives of our conference presentations, posters, and pamphlets.

### FlyBase Twitter and the “Tweetorial” project

The FlyBase Twitter account (@FlyBaseDotOrg) shares news about FlyBase updates and other items of interest to the Drosophila research community. In 2017, we began composing Twitter-based tutorials, colloquially called “Tweetorials,” which document new or improved FlyBase tools, reports, features, and datasets. The intent behind the Tweetorial project is to provide concise, image-heavy documentation of a specific topic in a shareable format that is faster and less labor-intensive to produce than video tutorials. From 2018 onwards, Tweetorials are tagged with “#FlyBaseTweetorial” and one or more topic-specific hashtags, rendering them searchable. We have added instructions on searching Twitter, as well as a complete list of FlyBase hashtags, to the FlyBase Wiki: https://wiki.flybase.org/wiki/FlyBase:Tweetorial, last accessed 03 March 2022. Resources permitting, the @FlyBaseDotOrg Tweetorial archive could form the basis for an image-enhanced set of help documents hosted within the FlyBase Wiki (see below).

### User documentation and the FlyBase Wiki

The FlyBase site contains a vast array of tools and reports, and it can be overwhelming to find and understand everything on the site. Fortunately, multiple options are available to help navigate FlyBase. Beyond the Tweetorials noted above, and the video tutorials hosted on YouTube (https://www.youtube.com/c/flybasetv), extensive documentation has been written on almost every aspect of the site at the FlyBase Wiki (https://wiki.flybase.org, last accessed 03 March 2022). There, one can find instructions covering all the major tools for finding and analyzing FlyBase data, field by field descriptions for each of the data type-specific FlyBase reports, guidelines to nomenclature and the curation of FlyBase data, instructions for authors, and much more.

There are two ways to access the Help documentation from almost every FlyBase page. For most tools or data report pages, one can generally find links to documentation specific to that tool or type of data report from within the page, usually in the top right hand corner of the page, may be in the form of a “Help” button, a “?” icon, or a help link in text. The second approach is to access Help information from the dropdown menu bar present at the top of each FlyBase page. The menu includes links to topic-specific help as well as a link to the “Help Index”, a compilation of links to nearly all of the FlyBase Help Documents.

### Help mail

FlyBase greatly appreciates feedback from users to help improve the database. To send questions or suggestions, use the “Contact FlyBase” links that can be found at the bottom of the Help dropdown menu and at the bottom of every regular FlyBase page. FlyBase staff monitor help emails, and queries will generally be answered by someone with appropriate expertise within three days. We are eager to help users find the information they are looking for in FlyBase, answer questions about the diverse tools in FlyBase, or address any other concerns. By letting us know what you find confusing or difficult to find, you offer useful feedback on aspects of the database that need improvement.

### FlyBase presentations and publications

An archive of posters, pamphlets, and conference presentations made by FlyBase is available from the FlyBase Presentations link in the “About” dropdown menu. These range in scope from broad overviews to detailed treatments of specific FlyBase features. A link to FlyBase publications can also be found in the “About” dropdown menu. Publications provide a more formal description of new data, tools, and reports in FlyBase, including examples and additional background information.

## Coming attractions and concluding remarks

FlyBase will continue to develop new tools and reports to make existing data and data types more accessible, to incorporate data reflecting emerging technologies and areas of research, to address user needs or requests, and to incorporate other data types that are gaining importance in model organism research.

### The genetic toolkit

Thanks to the introduction of the experimental tool data class, a large number of transgenic constructs in FlyBase now have a formalized description of their components, allowing researchers to robustly search for genetic reagents with particular characteristics. However, for those transgenic constructs that do not encode an experimental tool, the description of the encoded product is currently only captured as free text, precluding robust searching of this set of constructs. To make all transgenic constructs more accessible and interoperable, we plan to apply Sequence Ontology (SO; Eilbeck *et al.* 2005) terms to ensure all constructs have a formal description. This will allow researchers to identify transgenic constructs that encode either a wild-type or mutated gene product of a particular class (e.g. truncation, point mutation, internal deletion). Once all relevant constructs are annotated in this way, researchers will be able to easily compile a list of key genetic reagents that can be used as a “Toolkit” to study their gene of interest.

### scRNA-seq in FlyBase

Over the past few years, single-cell RNA sequencing (scRNA-seq) (Haque *et al.* 2017) has proved an invaluable tool in biomedical research. Many studies have already leveraged the technique in fruit flies (Karaiskos *et al.* 2017; Davie *et al.* 2018; Cho *et al.* 2020), and the number of scRNA-seq datasets will grow quickly in the coming years. We are therefore planning new features to facilitate access to *Drosophila* scRNA-seq data and metadata. Any scRNA-seq dataset obtained from *Drosophila* will be inventoried in FlyBase and linked to in multiple reports, allowing users to find, for example, all datasets from a given study, all datasets in which a given gene has been detected, or all datasets in which cells of a given cell type have been identified. In addition, the Gene report page will leverage scRNA-seq data to show cell-type-specific gene expression.

### Chemical curation

*Drosophila* increasingly serves as a useful model to study physiological effects of pharmacological and chemical treatments. These include potential environmental toxins, chemicals used for gene function discovery, and candidate therapeutics for treating human disease. FlyBase has begun to curate the chemicals used in studies of their effects on flies. The initial reports for a chemical will provide basic information including definition, synonyms, and biological roles pulled from two major databases, ChEBI (Hastings *et al.* 2016) from EMBL-EBI and PubChem (Kim *et al.* 2021) from NCBI. This will be paired with links to Drosophila publications using the chemical. We expect to eventually expand our annotation of chemicals, including incorporating them into disease and phenotype curation.

### Beyond FlyBase

We have taken this opportunity to expound upon the unique aspects of FlyBase, but we also have much in common with other MODs, including our commitment to FAIR (Findability, Accessibility, Interoperability, Reusability) principles (Wilkinson *et al.* 2019). For the last several years, FlyBase has actively contributed to the Alliance of Genome Resources, a collaborative web portal that gathers and harmonizes data from several model organism resources and the GO Consortium (Alliance of Genome Resources Consortium 2020). The integration of information across MODs facilitates cross-species analyses, improves access to data from many sources, and ultimately fosters collaboration across research communities.
